# Observations on Convulsion, as a Consequence of Disease, Illustrated by Cases

**Published:** 1810-05

**Authors:** John Howship

**Affiliations:** Surgeon


*Observations on Convulsion, as a Consequence of Disease, illustrated by Cases.* By Mr. John Howship, Surgeon.

No part of the animal economy has been so frequently the subject of fanciful speculation as the brain; an organ, the general purposes and offices of which may be deduced from the consideration of its situation and connections. Thus far we may have proceeded, but no further; for whenever we attempt to go beyond this line, and enter into the supposed manner in which the material and immaterial agencies operate reciprocally upon each other, in those various movements of the animal machine, in which volition is concerned, we soon find ourselves surrounded on every side by a maze of perplexity, where every fancied advance only proves how readily we deceive ourselves, and where even conjecture cannot find the least plausible foundation.

In considering the circumstances that indicate the due performance of the functions of the brain, in its healthy condition, and contrasting these with the numerous train of symptoms by which the presence of disease is pointed out, the most embarrassing conviction is, that we have repeatedly known that in cases where before death, every external sign of derangement in the structure of the brain has existed, examination afterward has decidedly proved the non-existence of any visible alteration, either in the structure, or even actions of the organ; and on the other hand, where no external mark or sign of particular disease has been observed to occur in the life-time of the patient, the most extensive state of disease in some part of the brain has been after death detected. It is our first duty to improve upon what we know, and the most important conclusion that can be drawn from such considerations as the above, certainly is this, that as the difficulties which here interrupt our progress are in their nature almost insurmountable, the ardour of our pursuit should be still further quickened; and that as the impediments to be removed are in themselves very considerable, so should our perseverance be taught to increase, until it shall rise even superior to every difficulty.

The treatment of convulsive disorders, supposed to depend on some derangement either in the structure or the functions of the sensorium, has at all times necessarily been governed according to the particular theory of the disease and its phenomena, that have been preferred by the

 practitioner.

practitioner. Upon this he has reasoned, and upon this he has built his practice, and in many, or in few instances, it has still been attended with success. In many cases of such disease terminating fatally, it has been proved by dissection, that a general state of convulsion has been produced and kept up in the habit, by the growth of some diseased substance within the cavity of the cranium. In other cases again, the same series of symptoms have arisen, and after continuing for a given length of time, they have disappeared, and from the good fortune of the patient in rec vering, the true result of the disease has never been known. Here is a door opened to conjecture, and from the fallacy of reasoning in such obscure inquiries, it is necessary that every opinion should be formed cautiously.

We have known of instances where tumours have formed within the skull, and where, as the disease increased, the oppressed portion of the brain has been from time to time removed from the situation of the tumour, by the action of the absorbent vessels; this action being set up as a provision, in order that by the sacrifice of a small part to the good of the whole, the circulation and functions of the general mass of the brain should still be maintained in all that undiminished freedom essential to the health and activity of the body.

We have seen instances, where not one, but many tumours, some of very considerable size, and probably of scrofulous nature, have been produced upon every part of the dura matral covering of the brain, and even in such a case as this, from the persevering action of the absorbent vessels in and about the cortical substance of the brain, the removal of the one material has kept pace with the deposition of the other, so that the functions of the sensorium were to a certain degree still carrying on, and life was still prolonged, until, from the continuing increase of the disease, the condition of the brain became at length so feeble and imperfect, that it could no longer impart the energies essential to life, and the complaint then soon destroyed.

In the consideration of the most proper treatment in epilepsy, as being one species of convulsion, very great diversity of opinion has prevailed; one class in the profession maintaining that it is to be cured only by diminishing and lowering the actions and powers of the constitution, and by withholding all those medicines and applications that can in any way produce excitement, either in the vascular or nervous system; while another class, that depletion is bad, inasmuch as convulsion arises as the consequence of an irritable ble state of fibre, and that whatever diminishes strength, increases mobility, and thereby confirms the very disorder which we intend to remove. Upon the same principle, that of allaying the increased irritability in the habit, a regular course of opiate medicines, under prudent conduct, is the most advantageous practice of any.

Upon both sides of this question, much has been said, and indeed the arguments brought forward in favour of each practice have been so ingeniously supported, and so powerfully contended for, that it becomes very difficult to say positively which, or if either of them, are altogether wrong; probably, with certain limitations, each may be right and proper in particular examples of these diseases. At all events, this conclusion must be just, and is at the same time very important, that while so much obscurity still dwells upon this branch of medical study, while our acquaintance is still so partial with most of the disordered actions to which the brain is subject, it is evident, that the most secure and firm foundation that can be laid for the advancement of our knowledge in these complaints, will be secured by our watching carefully, and setting down correctly, all the circumstantial histories that can be traced at the sick bed-side; if this task is performed with fidelity and truth, our stock of useful information must increase, in spite of every obstacle to its growth, and it will increase by the heaping up of a store of the most valuable of all materials.

---

### *A Case in which Convulsion, connected with great Constitutional Debility, was treated by the depleting System.*

Mrs. Cowan, the wife of a soldier, in the 82d regiment of foot, a very small and delicate figure of a woman, 28 years of age; from a combination of the most distressing incidents was exposed with an infant at the breast, for several days journey, in the severe winter of 1808. She had to pass through Lincolnshire, at a time, when the inclemency of the season had left the roads covered several feet deep in snow. This poor woman was several times, while journeying, so deep in the snow, that she very much doubted if she could ever get through it. Just before this, she had suffered shipwreck, and at the time when the vessel struck upon the rocks, she received so severe a shock from the alarm, that it was with difficulty the humane exertions of those around prevented her from fainting. Since that time, she said she never had been exactly well in her head. While on the journey, her peril was so great, that

 she she was sometimes obliged to lay down her little infant for a time upon the snow, in order that her exertions might be made with more freedom to extricate herself.

She reached however the town of Scarborough in Yorkshire, the place to which she was going, and being restored to her husband and family, she made no particular complaint for some time after. Her disorder then seemed to be deficient strength in the circulation and animal powers. The pulse was extremely small, low, and slow; the skin cold and very pale; the tongue clean and moist; the appetite very trifling. There was some head-ach, and she complained that she could not rest well at night, but was without refreshing sleep.

In order to restore, if possible, the proper balance of the circulation, and assist in the re-establishment of the external heat, very minute proportions of the tartarized antimony were directed in solution, to be frequently taken; and the patient was requested to avoid those constant personal exertions upon which the neatness, and indeed the existence of her family depended. This request was urged from the necessity of her own situation, but it was a desire with which it was ill in her power to comply. Although exceedingly feeble, she still continued to scour, wash, and do all the work of her family.

On the morning of Saturday, January the 14th, 1809, after very great exhaustion from labour, she fell suddenly down while passing over the step of her own door; a person who was near her, heard the fall, ran to her assistance, and found that she was entirely insensible. She was, as soon as possible, carried back to her bed, upon which she lay the whole of the forenoon; she was apparently in a tranquil state, as if asleep; respiration was continued with great freedom and regularity. The face was pale, sunk, and cold; frequent slight convulsive tremors took place, and these occasionally returned, for the space of two hours, when they altogether disappeared.

In the evening the pulse was at 70, and the tremors had returned with occasionally rather violent contractions of the muscles of the limbs, at which periods, the pulse became perceptibly harder than before. In about half an hour, however, she came to herself, soon after the spasms had ceased; she opened her eyes, and complained of great and shocking pain in her head, attended with terrible shooting darts of pain passing through the eye-balls; she could not bear a candle to be brought near the bed, from its aggravating the painful state of her eyes. With the above above symptoms, she now mentioned her having a constant roaring noise in her ears as if it thundered; her countenance was still sunk, and her complection paleness itself. Yet from her symptoms, notwithstanding her excessive weakness, it was pointed out to her that she must lose blood; the proposal somewhat startled her, but she made no objection to what was advised. The arm was bound up for some minutes before, with some difficulty, a vein was perceived; it was sufficiently evident to the touch, but so small, and so far below the surface, that it was not discernable to the eye. This vein was opened, and about three ounces of blood were drawn; a vein in the other arm was next opened under the same circumstances, and with the same effect; so that by this operation, she lost about six ounces of blood; this to her was a considerable quantity, and she found herself materially relieved; she said she was much better, but that she was still "very bad."

An hour after she had been bled, while laying very comfortably warm in bed, she was suddenly seized with a rigor and shivering, exactly resembling the first stage of an ague fit. The pulse was at this time very small but hard; and the skin very pale; she was, soon after this, sensible; and said again, that she was "very bad." This state of tremor did not perfectly subside before half an hour had passed over, when it ceased. Her head was now directed to be shaved, and covered with a large blister; after this application had been made, she was ordered to take thirty drops of the tincture of opium in a draught, and this was repeated soon afterwards, which procured her some sleep.

Sunday morning, she was something better; the tartarised infusion of senna was directed, and repeated till it produced two motions; towards the evening of this day she found herself much better, but on a sudden and very unexpectedly, her eyes were closed; her limbs became rigid; and she was shaken by a convulsion decidedly more violent than ever. Her body was at some moments raised up from the bed by the violence of the fit. At this time her pulse was seventy-two, and small. The convulsion subsiding, she soon came to herself, and upon being spoken to, said she had still the same loud voice in her ears, and the same extreme sensibility to light. In half an hour more, she was much better, and spoke quite distinctly and rationally, and explained that the pain in her head was very much relieved. Before the bleeding, this was of a dull, stupid, heavy, and oppressive kind, but it was now of another

 character, character, being sharp, and frequently darting about the brain. The most important point of relief, however, as she herself expressed it, was, that now her mind and judgment was much relieved, and more clear than before. There was no heat about the skin, nor the least thirst; she this day took the saline mixture frequently, with ten drops of the tincture of opium in each dose.

On Monday morning, January the 16th, there was occasional light wandering and tendency to delirium; she said upon being examined, that she had now no noise in her ears, nor the least pain whatever in her head, but was in every respect quite easy. The pulse had risen to 84; the saline mixture as the day before, was continued.

In the afternoon she was perfectly sensible, without even the least disposition to delirium; but precisely at the same hour as the day before, she was seized with slight spasms, the eyes now open and fixed in their sockets; in this state she was perfectly insensible and very pale; she soon after recovered again.

In the evening she was quite sensible and clear; the pulse being soft and regular, at 75; the skin moist and perspirable. A swelling of the face has come on during the day, productive of much pain, and very tender. The light is no longer distressing to the eyes; the roaring sound, as of thunder, is no longer heard; the pain in the head nearly gone.

This morning she said that she felt "ill" all over her, but this evening she was much better, having neither heat nor thirst. She was directed through the day to go on as before with her saline medicines, and to have her feet immersed in warm water at bed-time.

Tuesday morning. She had passed a good night, and slept very well; and upon the whole was very much improved. At noon the light delirium returned for a time; her mind wandered, as it were, imperceptibly; without the least sympathetic change in any other respect. In about an hour this subsided, without having been attended with any degree of spasm, or the least general indisposition. During the course of the day she complained several times of the pain in her face.

Wednesday, January the 18th, she was very restless; got no sleep the night before; she described a sensation of pain very distressing about the heart, "as if something was gnawing it." The bowels were very regular; the saline mixtures as before were continued; and a blister directed to be laid upon the pit of the stomach.

From

From this time she continued to improve daily, her appetite returned in some measure; so that by taking nourishment in light forms she was hourly gaining ground.

This state of convalescence continued till the Wednesday following, January the 25th, when she suddenly fell off again. On the day before, she had felt some little pain in her head, but did not consider it worth mentioning; but on this day it was much worse, indeed, so severe, that she could scarcely sit up. In the evening she had the same pain in an aggravated degree, add to which she could not now bear the light to be brought near her eyes. Upon examining her wrist, it was found that the pulse had again sunk down as before her convalescence, from 80 in the minute to 70; the pulsation was rather hard than not; she complained of sickness, and several times urged without effect, to relieve her stomach by vomiting; she had no thirst; her skin was natural, and bowels regular. She was ordered the saline mixture every hour, and to have her feet immersed in hot water at bed-time.

On the next day, Thursday, she was again much better, and was nearly relieved from the pain in her head. She was reduced to a state of excessive lowness and debility, but it was necessary to be very cautious, in the adoption of any measures for the recovery of her strength; for it should have been before mentioned, that during the week of her improvement, and while she was perfectly free from her complaints, she had been allowed to take in very minute proportions, a light infusion of bark, and it was to this circumstance that her present relapse was attributed.

On Thursday evening she was ordered to take an ounce of the castor oil, to have her feet bathed, and to take her salines every hour during the night.

From this time she continued to mend uniformly, till, on Sunday, February the 5th, she was seized while scouring out the room, with terrible shivering, so severe, as if cold water had been pouring in a stream down her back, and this was attended with very excruciating pain in her head, and in all her limbs pains also.

By confinement, and a return to her former treatment for a few days, she was again relieved, and advised to be more cautious for the future, in exposing herself to cold. For some time after this illness, so great was her general state of debility, that she complained of her legs swelling towards night.

For the improvement of her appetite, which was still very deficient, an infusion of the flowers of chamomile was was recommended, this not being so objectionable as the more powerful tonics. She for some time persevered in the use of this medicine, and gradually recovered her health altogether.

*Mill Street, Hanover Square,*
*March* 14, 1810.

( To be continued. )

